# Supplementary material for: Causal Relationship between Aging and Anorexia Nervosa: A White-Matter-Microstructure-Mediated Mendelian Randomization Analysis
Source: Biomedicines. 2024 Aug 16;12(8):1874. doi: 10.3390/biomedicines12081874 (PMC11351342; doi:10.3390/biomedicines12081874)
Supplement: Supplementary file 1 [file biomedicines-12-01874-s001.zip › Supplementary Figure/Figure S4/Singe SNP plot of MR analysis for TL to ubm-b-1625.pdf]

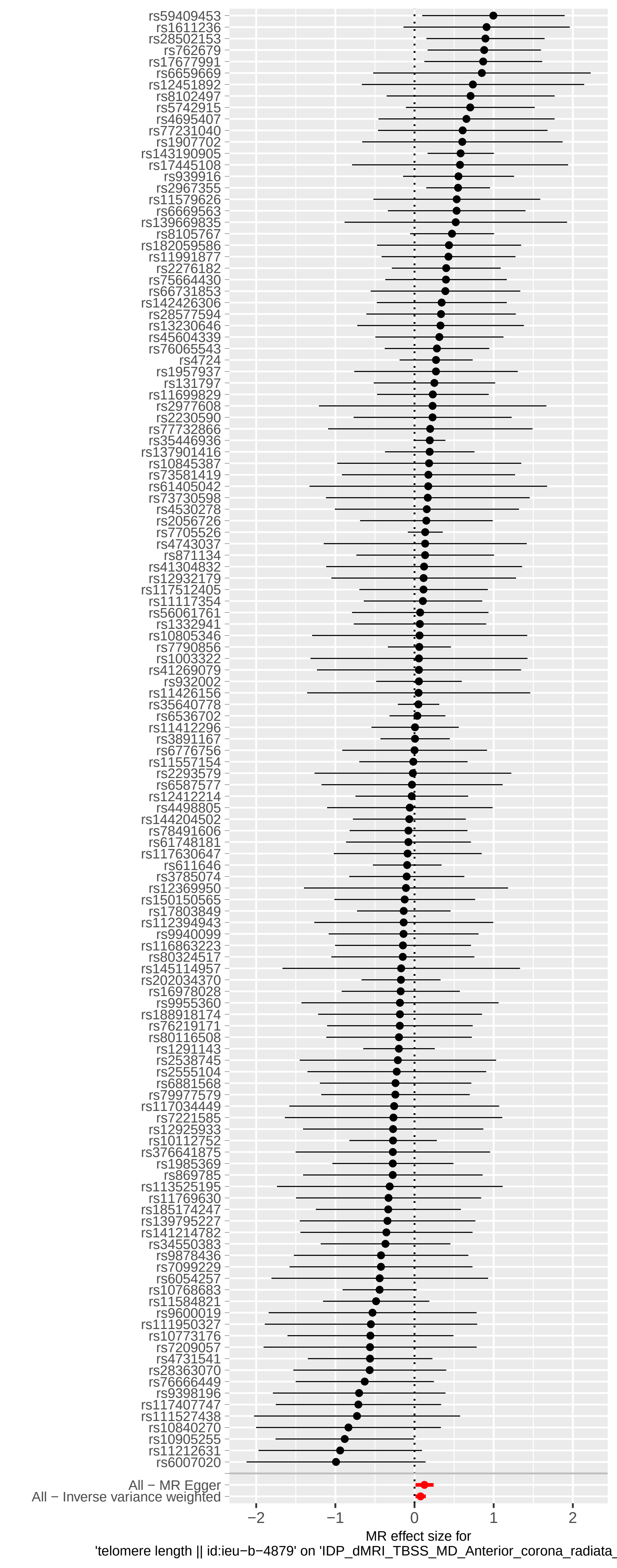
rs59409453
rs1611236
rs28502153
rs762679
rs17677991
rs6659669
rs12451892
rs8102497
rs5742915
rs4695407
rs77231040
rs1907702
rs143190905
rs17445108
rs939916
rs2967355
rs11579626
rs6669563
rs139669835
rs8105767
rs182059586
rs11991877
rs2276182
rs75664430
rs66731853
rs142426306
rs28577594
rs13230646
rs45604339
rs76065543
rs4724
rs1957937
rs131797
rs11699829
rs2977608
rs2230590
rs77732866
rs35446936
rs137901416
rs10845387
rs73581419
rs61405042
rs73730598
rs4530278
rs2056726
rs7705526
rs4743037
rs871134
rs41304832
rs12932179
rs117512405
rs11117354
rs56061761
rs1332941
rs10805346
rs7790856
rs1003322
rs41269079
rs932002
rs11426156
rs35640778
rs6536702
rs11412296
rs3891167
rs6776756
rs11557154
rs2293579
rs6587577
rs12412214
rs4498805
rs144204502
rs78491606
rs61748181
rs117630647
rs611646
rs3785074
rs12369950
rs150150565
rs17803849
rs112394943
rs9940099
rs116863223
rs80324517
rs145114957
rs202034370
rs16978028
rs9955360
rs188918174
rs76219171
rs80116508
rs1291143
rs2538745
rs2555104
rs6881568
rs79977579
rs117034449
rs7221585
rs12925933
rs10112752
rs376641875
rs1985369
rs869785
rs113525195
rs11769630
rs185174247
rs139795227
rs141214782
rs34550383
rs9878436
rs7099229
rs6054257
rs10768683
rs11584821
rs9600019
rs111950327
rs10773176
rs7209057
rs4731541
rs28363070
rs76666449
rs9398196
rs117407747
rs111527438
rs10840270
rs10905255
rs11212631
rs6007020
All − MR Egger
All − Inverse variance weighted
−2
−1
0
1
2
MR effect size for
'telomere length || id:ieu−b−4879' on 'IDP_dMRI_TBSS_MD_Anterior_corona_radiata_
